# Study on follicular characteristics, hormonal and biochemical profile in norgestomet+PMSG treated acyclic buffaloes

**Published:** 2016

**Authors:** A. Jerome, S. K. Srivastava, R. K. Sharma

**Affiliations:** 1 Animal Physiology and Reproduction Division, ICAR-Central Institute for Research on Buffaloes, Haryana, 125001, India;; 2 Animal Reproduction Division, ICAR-Indian Veterinary Research Institute, Bareilly, Uttar Pradesh, 243122, India

**Keywords:** Acyclicity, Buffalo, Follicular dynamics, Norgestomet, PMSG

## Abstract

This research was conducted to study the follicular dynamics, hormonal, biochemical profile and fertility response in acyclic and norgestomet+PMSG treated acyclic buffaloes in summer. The study animals were divided into two groups: group I [control (n=8): no treatment] and II [treatment group (n=15)]. In group II, seven animals were used for follicular biochemical and hormonal profile and eight animals for fertility studies following Crestar^®^ (Intervet, France) treatment (day 0: Crestar^®^ insertion; day 8: 500 IU PMSG; day 9: Crestar^®^ removal; day 11 AI). Follicular fluid stradiol (E2) and progesterone (P4) in acyclic and pre-ovulatory follicle in study groups was significantly (P<0.01) higher than peripheral level. Peripheral E2 concentration, during pre-ovulatory period in group II was higher (P<0.05) than group I. Significant correlation between serum and follicular E2 was deduced (r=0.888; P<0.01) as significant difference in serum cholesterol content was shown between groups. Lower follicular total protein (P<0.05) in acyclic animals and higher follicular glucose (P<0.05) in treated group were concluded. Significant correlation (r=-0.770; P<0.05) was observed between follicular cholesterol and triglycerides. Follicular characteristics, post PMSG administration, differed significantly (0.83 ± 0.20 vs 1.32 ± 0.12; P<0.01) in all buffaloes exhibiting estrus, out of which four conceived. In conclusion, follicular hormonal and biochemical profile exhibits alteration in protein and glucose level between summer acyclic and treated buffaloes. However, peripheral E2 along with fertility response showed significant difference (P<0.01) between the study groups with significant correlation in E2, cholesterol and triglycerides between peripheral and follicular compartment.

## Introduction

Incidence of anestrus in buffaloes during summer varies between 36.6 and 59.5% (Singh et al., 1989 ▶; Das and Khan, 2010 ▶). Anestrus in buffaloes is attributed to poor feeding, stress and season (Zicarelli, 1997 ▶; Qureshi et al., 2002 ▶; Borghese, 2005 ▶). During summer, majority of buffaloes show silent estrus (15-73%) and/or estrus with shorter duration (De Rensis and Scaramuzzi, 2003 ▶; Barile, 2005 ▶; El-Wishy, 2007; Das and Khan, 2010 ▶). Seasonality and lack of nutrition leads to derangement of hormones’ secretion pattern in cattle and buffaloes (Qureshi et al., 2000 ▶; Khodaei-Motlagh et al., 2011 ▶). Investigations have shown that buffalo follicular hormonal profile varies with follicular size, stage of estrous cycle, cyclicity and infection (Thangavel 2004 ▶; Marai and Haeeb, 2010 ▶; Khan et al., 2011 ▶, 2012; Baki Acar et al., 2013). Also, E2:P4 is altered by cyclicity (Arshad et al., 2005 ▶; Alkalby et al., 2012 ▶; Varughese et al., 2014 ▶). At present, little (Kumar et al., 2015 ▶) or no information on corresponding serum hormonal and biochemical profile is available, as these investigations, based on slaughter-house specimens, fail to depict follicular characteristics as well correlation between follicular and peripheral hormone and biochemical milieu in summer acyclic buffaloes. With metabolic changes and acyclicity follicular ﬂuid biochemical composition changes (Khan et al., 2011 ▶; El-Shahat and Kandil, 2012 ▶), but the exact milieu of follicular constituents during summer acyclicity, if there is any post estrus induction in summer anestrous buffaloes needs to be studied for elucidating the role and mechanism of follicular hormone and biochemical profile in acyclic and estrus induced buffaloes.

Anestrous buffaloes, though treated with several hormonal regimens (Barile, 2005 ▶; De Rensis and Lopez-Gatius, 2007 ▶) have shown that P4 based treatment regimens were more effective for inducing cyclicity in summer anoestrus buffaloes (Barile et al., 2001 ▶; Neglia et al., 2003 ▶; Singh, 2003 ▶). Crestar^®^ results in higher estrus induction (>80%) and conception rates of 45-60% in cattle and buffaloes (Ozyurtlu et al., 2009 ▶; Dodamani et al., 2011 ▶; Chaudhari et al., 2012 ▶; Pandey et al., 2013 ▶; Chaudhary et al., 2015 ▶). Studies on follicular dynamics following norgestomet+PMSG (pregnant mare serum gonadotrophin) treatment in buffaloes have been documented (Rohilla et al., 2005 ▶; Malik et al., 2010 ▶, 2011). However, studies on follicular microenvironment vis-à-vis peripheral hormonal, biochemical profile following norgestomet+PMSG treatment in acyclic buffaloes in summer have not been studied, so far. Furthermore, studies pertinent to alterations of follicular and peripheral hormonal and biochemical constituents following estrus induction are lacking. Considering this paucity, the present study was designed to test the hypothesis that any follicular and peripheral hormonal and biochemical profile along with follicular dynamics in summer acyclic buffaloes following estrus induction treatment using norgestomet+PMSG hormone regimen.

## Materials and Methods

**Location of study and animal management**

 The study was conducted at ICAR-Central Institute for Research on Buffaloes, Hisar, located in Haryana State of India. It is located 212 m above mean sea level, latitude is 29.17 North and longitude is 75.72 East. This study was conducted during summer (May to August) on twenty three post-partum (>90 days) anoestrus buffaloes aged 4.5-6.5 years with body weight 400-550 kg with BCS>3 (Edmonson *et al*., 1989). All animals were managed under semi-intensive system and fed *ad libitum* rations containing green fodder, wheat straw (2-2.5 kg), concentrate feed @ 6 kg/animal/day along with mineral mixture. All the experimental procedures were carried out with the approval of Institutional Animal Ethical Committee.

**Ovarian cyclicity monitoring and estrus induction**

Repeated alternate day transrectal ultrasonographic examinations of ovarian activity starting from two weeks before the start of study was carried out in all buffaloes using a B-mode ultrasound scanner (Toshiba, SSA 220, JustVision) equipped with an intraoperative 7.0 MHz microconvex transducer. Buffaloes, with no corpus luteum, at an interval of 10 days, were adjudged as acyclic. During ultrasonographic examination, each ovary was scanned in several planes with the transducer along the ovarian surface. Position and size of follicles were traced at each scanning and relative position of follicles to facilitate sequential evaluation of follicular turnover was recorded. Ovulation was determined by the disappearance of a large follicle (>12 mm) and subsequent appearance of a corresponding corpus luteum at the exact location in the same ovary as described by Sharma et al. (2012) ▶. The animals under study were divided into two groups viz. group I (control) and II (treatment). In group I, eight acyclic buffaloes, with no treatment, were used for studying follicular biochemical, hormonal profile and fertility status. In group II, fifteen acyclic buffaloes were used with seven animals for follicular biochemical and hormonal profile and eight animals for fertility studies following Crestar^®^ (Intervet, France) treatment (day 0: Crestar^®^ implant insertion; day 8: 500 IU PMSG injection; day 9: Crestar^®^ implant removal; day 11 AI was tried). Crestar contains 3 mg norgestomet (synthetic progesterone) as active ingredient used for induction of estrus in cattle and buffaloes. In the present study, cyclicity was deduced by the presence or absence of corpus luteum by transrectal ultrasonographic examination 10 days apart.

**Follicular sampling**

*In situ* follicular fluid (follicles >10 mm in diameter) sampling was done in both groups following standard procedures as described by Pieterse et al. (1988). ▶ Follicular sampling was done 48 h after PMSG treatment in treated groups. Along with follicular sampling, blood (10 ml) was collected for hormone (E2 and P4) estimation in serum. Before each follicular fluid aspiration, epidural anesthesia was performed using 6 ml of lignocaine hydrochloride 2% (Xylocaine 2%, Astra IDL, India) between the first and second coccygeal vertebrae. After this, follicle is visualized by ultrasound equipment (Esaote, Aquila Vet) connected to a 7.5 MHz microconvex array transvaginal transducer equipped using 18 G needle guide and connected to a regulated vacuum pump (K-MAR-5100, Cook IVF Co., Australia). The contents of the follicles with a diameter of >10 mm were aspirated with pressure of 50 mm Hg using a vacuum pump into a sterile 15 ml tube.

**Hormone and biochemical analysis**

Hormones (E2, P4) estimation in follicles and serum was done following ELISA protocol (Cusa Biotech. Co. Ltd., China). The intra and inter-assay coefficient of variation was <8 and <10%, respectively. Blood samples (10 ml) were collected on day of follicular aspiration from both groups in BD vacutainer^®^ serum tubes. Serum was harvested by centrifugation of the vacutainer tubes at 3000 rpm for 15 min and collected serum was stored at -20°C for hormone estimation. Simultaneously, blood sampling was done during follicular fluid aspiration for estimation of serum hormone (E2, P4) by using ELISA kit (Cusa Biotech. Co. Ltd., China). The sensitivity of P4 and E2 was 0.12 ng/ml, and 0.75 pg/ml, respectively. The intra and inter-assay coefficient of variation was <8 and <10%, respectively. Follicular and serum biochemical parameters viz. total protein, cholesterol, triglycerides and glucose were analyzed in Coralyzer-200 (Tulip Diagnostics. Pvt. Ltd., India) following kits’ protocols (Coral Clinical Systems Pvt. Ltd., India).

**Fertility studies**

Estrus detection was carried out towards the end of treatment with visual signs viz. estrous behavior, discharge, urination and vulval swelling, i.e. in the morning and evening. Buffaloes in heat were inseminated with 0.25 ml frozen semen straw following AM-PM method and pregnancy was confirmed by day 30 using ultrasonography.

**Statistical analysis**

Statistical analysis was carried out as per Snedecor and Cochran (1989) ▶ using SAS software. Repeated measures ANOVA was done to determine the difference in means within days and across groups with Tukey’s Post Hoc test. The differences in mean were analyzed by Mann-Whitney multiple comparison test. Correlation between serum and follicular hormonal and biochemical parameters was carried out by Pearson’s correlation co-efficient. Data represented as mean±SE and considered significant at P<0.05.

## Results

Follicular characteristics (growth rate and ovulation), estrus signs and response between the study groups are shown in Table 1. Though, no significant difference in follicular characteristics between groups prior to PMSG was observed, post PMSG, there was a significant difference (P<0.01) in follicular growth rate and size in treated group. Furthermore, all treated buffaloes exhibited estrus signs with four buffaloes conceiving as compared to none in control group. Follicular E2 and P4 were significantly (P<0.01) higher than serum irres-pective of study groups. Serum E2 in treated group was significantly higher (P<0.05) during pre-ovulatory period as compared to control group (Table 2). All treated buffaloes showed estrus signs (frequent urination, clear vaginal discharge and vulval tumification) prior to follicular aspiration. Correlation between serum and follicular hormone profile in groups showed significant correlation was found between serum and follicular E2 (r=0.888; P<0.01) (Table 3). Significant difference (P<0.05) in serum cholesterol was deduced between the control and treated group but not in follicular cholesterol. Triglycerides (follicular and serum) did not differ significantly (P>0.05) between and within groups. Lower follicular protein in acyclic animals and higher follicular glucose in treated buffaloes was deduced in the present study (Table 4). Correlation between serum and follicular biochemical constituents showed a significant (r=0.770; P<0.05) correlation between follicular choles-terol and triglycerides (Table 5).

**Table 1:** Follicular dynamics and fertility response in acyclic and treated buffaloes

Characteristics	Group I (n=8)	Group II (n=8)
**Follicular characteristics**		
Follicle >10 (mm) on day 0	4^a^	5^a^
Follicle <10 (mm) on day 0	5^a^	3^a^
Diameter of largest follicle (mm) at start of treatment	10.08 ± 0.58^a^ (9.5-10.65)	10.42 ± 0.36^a^ (10.05-10.80)
**Follicular growth rate (mm/day)**		
Before pregnant mare serum gonadotrophin (PMSG)	-	0.83 ± 0.20^a^
After pregnant mare serum gonadotrophin (PMSG)	-	1.32 ± 0.12^b^
Diameter of largest follicle at aspiration/ovulation (mm)	10.66 ± 0.88^a^ (9.5-11.55)	14.21 ± 0.32^b^ (13.5-14.5)
Follicular fluid (ml)	0.5 ± 0.05^a^ (0.45-0.55)	0.8 ± 0.05^a^ (0.75-0.85)
**Fertility parameters**		
Animal induced to estrus	0^a^ (0%)	8^b^ (100%)
Animal showing vulvar swelling	0^a^ (0%)	8^b^ (100%)
Animals showing mucus discharge and uterine tone	0^a^ (0%)	8^b^ (100%)
No. of animals ovulated	0^a^ (0%)	5^b^ (62.5%)
Time of ovulation (h) after end of treatment	-	36 ± 1.23

n: Number of animals or observations, values in a row with different superscript differ significantly (P<0.01)

**Table 2:** Follicular and serum hormone profile in study groups

Parameters	Group I (n=8)			Group II (n=7)	
	Serum	Follicle		Serum	Follicle
Estradiol (ng/ml)	0.0029 ± 0.01^a^	5.6 ± 0.33^b^*		0.52 ± 0.013^c^	3.27 ± 0.32^b^*
Progesterone (ng/ml)	0.30 ± 0.84^d^	5.36 ± 0.53^e^*		0.48 ± 0.08^d^	3.42 ± 0.42^e^*

n: Number of animals or observations, values expressed as mean±SE, values in a row for each hormone within and between group with different superscripts differ significantly

* (P<0.01)

**Table 3:** Correlation between serum and follicular hormonal profile in group I and II

Parameters		Group I					Group II			
		Progesterone		Estradiol			Progesterone		Estradiol	
		Follicle		Serum	Follicle		Follicle		Serum	Follicle
Progesterone	Serum	0.017		0.271	0.193		-0.152		-0.535	0.356
	Follicle	-		0.278	0.310		-		0.298	-0.542
Estradiol	Serum	-		-	0.888**		-		-	-0.742

** P<0.01

**Table 4:** Biochemical profile in pre-ovulatory follicle vis-à-vis serum in acyclic and Crestar treated buffaloes

Parameters	Group				
	Group I (n=8)			Group II (n=7)		
	Serum	Follicle		Serum	Follicle	
Triglyceride (mg/dl)	39.91 ± 0.94^a^	39.70 ± 3.25^a^		41.72 ± 1.40^a^	42.48 ± 1.04^a^	
Cholesterol (mg/dl)	49.90 ± 3.83^a^	113.07 ± 7.19^b^*		95.28 ± 6.87^c^	115.94 ± 4.50^b^*	
Total protein (g/dl)	6.58 ± 0.38^a^	4.94 ± 0.36^b^*		6.64 ± 0.60^a^	7.7 ± 0.31^a^	
Glucose (mg/dl)	58.42 ± 6.17^a^	59 ± 3.53^a^		54.68 ± 2.51^a^	74.3 ± 2.02*^b^	

n: Number of animals or observations, values expressed as mean±SE, values in a row within and between groups with different superscript differ significantly

* (P<0.05)

**Table 5:** Correlation between serum and follicular biochemical parameters in group I and II

Parameters		Group I							Group II						
		Cholesterol	Triglyceride		Total protein		Glucose		Cholesterol	Triglyceride		Total protein		Glucose	
		Serum	Follicle	Serum	Follicle	Serum	Follicle	Serum	Serum	Follicle	Serum	Follicle	Serum	Follicle	Serum
Cholesterol	Follicle	-0.572	-0.770*	-0.384	-0.621	0.188	0.186	0.053	-0.967	-0.688	0.018	-0.133	-0.201	-0.462	0.438
	Serum	-	0.466	0.390	0.718	0.348	-0.391	0.095	-	0.843	-0.109	0.429	0.938	0.222	-0.994
Triglyceride	Follicle	-	-	0.096	0.659	0.122	-0.258	0.134	-	-	-0.546	0.767	0.279	-0.337	-0.370
	Serum	-	-	-	0.171	0.254	-0.251	0.458	-	-	-	-0.457	0.489	0.945	0.690
Total protein	Follicle	-	-	-	-	0.082	-0.150	0.375	-	-	-	-	0.533	-0.786	0.158
	Serum	-	-	-	-	-	0.119	0.108	-	-	-	-	-	-0.130	0.669
Glucose	Follicle	-	-	-	-	-	-	-0.436	-	-	-	-	-	-	-0.327

* P<0.05 level

## Discussion

Follicular hormonal, biochemical profile and dynamics is deranged during acyclicity in buffaloes (Alkalby et al., 2012 ▶; Baki Acar et al., 2013; Kumar et al., 2015 ▶). This study documents the alterations in follicular dynamics along with hormonal, biochemical profile in acyclic and norgestomet+PMSG treated acyclic buffaloes in summer. In this study, follicular E2 and P4 were significantly higher than its corresponding peripheral concentration which was in consonance with Roth et al. (2001) ▶ and Khan et al. (2015), but in contrast with Khan et al. (2012) ▶ in buffaloes where negative follicular E2:P4 ratio was reported. This could be due to lack of health, nutritional and reproductive status of the buffaloes selected for the investigation. It is notable that in acyclic buffaloes, follicular hormonal prolife remained unaltered contrary to earlier findings (Khan et al., 2012 ▶). This can be attributed to the difference in study design as this study involved buffaloes maintained at uniform management conditions as compared to earlier slaughter-house based studies. It can be speculated that acyclicity might arise due to the absence of P4 priming rather than deranged follicular hormonal profile. Peripheral E2, during pre-ovulatory period was significantly higher in treated group due to the progressive growth of the pre-ovulatory follicle during the peri-estrus period following P4 priming. Significant correlation between serum and follicular E2 was in accordance with Varughese et al. (2014) ▶ and Kumar et al. (2015) ▶ in buffaloes. But, lack of correlation between follicular P4 and peripheral concentration or with E2, differing with Varughese et al. (2014) ▶ , can be attributed to difference in reproductive status. PMSG administration at the end of Crestar treatment had a direct effect on the progressive growth and ovulation of the follicle. But, this was not observed in acyclic buffaloes due to the lack of progesterone priming, even in the presence of dominant follicle. Progesterone priming by norgestomet up-regulates estrogen receptors at hypothalamaus and augments estrus behavior (Cerri et al., 2009 ▶; 2011; Wiltbank et al., 2011 ▶). This ascertains the importance of progesterone priming for estrus induction and ovulation. Follicular diameter and growth rate prior to PMSG treatment showed no difference between the groups as reported earlier (Badinga et al., 1993 ▶; Rohilla et al., 2005 ▶). But, post PMSG, follicular diameter and growth rate showed significant difference due to its folliculogenic activity by inducing the higher centers for enhanced follicular turnover, growth (Kumar et al., 2010 ▶; Fu et al., 2013 ▶), behavioral estrus (Singh et al., 2004 ▶; Malik et al., 2011 ▶). But, such effect was lacking in control group due to non-priming of progesterone and absence of stimulatory activity of PMSG (Rohilla et al., 2005 ▶).

With biochemical profile, triglycerides concentration was in conformity with Leroy et al. (2004) ▶ and Alkalby et al. (2012) ▶ in cattle and buffaloes respectively, but higher than Baki Acar et al. (2013) which could be due to species differences and nutrition. Significant difference was noticed in cholesterol concentration between serum and follicle in both groups as compared to earlier studies (Arshad et al., 2005 ▶; Alkalby et al., 2012 ▶) that can be due to nutritional status difference and sampling. Peripheral cholesterol level was similar to Arshad et al. (2005) ▶, but differed with Khan et al. (2012) at the follicular level, attributing to differences in study design, sampling, breed difference and nutrition. Significant negative correlation in follicular cholesterol and triglycerides were in agreement with Khan et al. (2011), attributing to its function of secondary energy source.

Higher peripheral cholesterol in treated group could be accredited to PMSG, resulting in enhanced synthesis cholesterol for E2 induced luteinizing hormone surge needed for ovulation. Significant low follicular total protein in control group as compared to serum was in conjunction with studies in buffaloes (Arshad et al., 2005 ▶; Alkalby et al., 2012 ▶). Though, it is evident that follicle protein remains consistent with peripheral level (Arshad et al., 2005 ▶; Abdellah et al., 2010 ▶). This discrepancy in protein level between the two com-partments can be due to the protein equilibrium main-tenance between serum and follicle compartments. Significant difference in glucose between the two compartments in treated group might be due to low metabolism of glucose in large follicles in treated group (Khan et al., 2012). Furthermore, high glucose levels in pre-ovulatory follicles might be due to the increased follicular growth facilitating nutrients across the blood-follicular barrier (Leroy et al., 2004 ▶; Khan et al., 2011 ▶). Higher estrus induction and conception rates in treated group, was in consonance with earlier reports in buffaloes (Nayak et al., 2009 ▶; Malik et al., 2010 ▶; Thangapandiyan et al., 2015 ▶). Norgestomet with PMSG act synergistically in inducing cyclicity (El-Fadaly et al., 1994 ▶; Lohan et al., 2001 ▶; Baruselli et al., 2004 ▶) by increasing the LH pulse frequency and its receptors with elevated E2 concentrations from pre-ovulatory (Garcia-Winder et al., 1987 ▶). This higher circulatory E2 level induces behavioural estrus (Singh et al., 2004 ▶; Malik et al., 2011 ▶) ascertaining the necessity of P4 priming of hypothalamus for behavioral estrus with stimulatory effect of PMSG in inducing follicle turnover (Wiltbank et al., 2011 ▶) with non-significant alterations in follicular hormonal milieu.

In summary, it is evident that follicular hormonal profile exhibits no alteration between acyclic and norgestomet+PMSG treated buffaloes, but serum E2 and follicular biochemical along with fertility response showed a significant difference between the study groups with significant correlation in E2, cholesterol and triglycerides between serum and follicular concentration.
